# A low temperature-adapted *Euglena gracilis* ecotype for outdoor biomass production in colder climates

**DOI:** 10.1128/aem.00195-26

**Published:** 2026-05-04

**Authors:** Shun Tamaki, Marumi Ishikawa, Toshihisa Nomura, Koji Yamada, Kengo Suzuki, Keiichi Mochida

**Affiliations:** 1 Bioproductivity Informatics Research Team, RIKEN Center for Sustainable Resource Science 98319, Yokohama, Japan; 2 Department of Biological Science, Faculty of Science and Engineering, Yasuda Women's University 13152 , Hiroshima, Japan; 3 R&D Center, Euglena Co., Ltd., Tokyo, Japan; 4 Microalgae Resource Upcycling Research Laboratory, RIKEN Baton Zone Program, Yokohama, Japan; 5 Kihara Institute for Biological Research, Yokohama City University 13155, Yokohama, Japan; 6 School of Information and Data Sciences, Nagasaki University 12961, Nagasaki, Japan; University of Milano-Bicocca, Milan, Italy

**Keywords:** *Euglena*, microalga, environmental adaptation, low temperature, lipid, paramylon, carotenoid

## Abstract

*Euglena gracilis* strain Z is currently used for industrial purposes such as the production of the polysaccharide paramylon, lipids, and carotenoids. This strain shows promise for sustainable bioproduction, but its growth is inhibited at temperatures below 20°C, limiting its geographic range for outdoor cultivation. Here, we discovered a new low temperature-adapted *E. gracilis* ecotype, strains min34 and min41, in freshwater from a northern prefecture of Japan. These strains were isolated during the participatory science project “Everyone’s Euglena Project.” Strain min41 thrives at 15°C, achieving cell densities and dry weights of 5.2- and 7.5-times higher than those of strain Z, respectively. Strain min34 also showed high growth ability at 15°C but was inferior to strain min41. Whereas strain Z exhibits reduced bioproduction capacity at 15°C (as evidenced by decreased paramylon, lipid, and carotenoid production), strain min41 maintains high bioproductivity at low temperature. Our findings underscore the rich genetic potential within *E. gracilis* for adaptation to diverse conditions and offer a valuable bioresource for exploring temperature-adapted systems, broadening the prospects for biomass production.

**IMPORTANCE:**

Outdoor cultivation of microorganisms for bioproduction is often limited by temperature. Industrially useful *E. gracilis* strain cannot grow well in colder climates, restricting their use in sustainable industries such as food and biofuel production. In this study, we identified a new low temperature-adapted strain of *E. gracilis* that can thrive and remain productive at 15°C. This strain could enable year-round biomass production in regions with colder weather, expanding the potential of environmentally friendly biomanufacturing. Our discovery also highlights the untapped natural diversity of this microorganism, which may hold the key to developing more resilient and efficient bioproduction systems.

## INTRODUCTION

*Euglena gracilis* is an industrially utilized microalga with the ability to capture and use CO_2_, thus enabling sustainable bioproduction. *E. gracilis* performs well in large-scale outdoor cultivation systems and can be used to produce paramylon, a β-1,3-glucan polysaccharide that can be converted into wax esters under hypoxic conditions (1, 2). Paramylon holds promise as a versatile polymer for industrial applications, and wax esters are suitable for use as bio-jet fuel or biodiesel (1, 3). Therefore, enhancing the efficiency of biomass production in *E. gracilis* is crucial for maximizing its industrial applications.

Although *E. gracilis* has garnered industrial attention for its use for sustainable bioproduction, large-scale cultivation of the alga is geographically and seasonally constrained due to its sensitivity to temperature fluctuations. Temperature stress in algal cells alters protein homeostasis, membrane fluidity, oxidative stress status, cellular metabolism, and the cell cycle (4, 5), resulting in decreased biomass productivity. Under mixotrophic conditions, the widely used *E. gracilis* strain Z (6) shows a significant reduction in growth rate at lower temperatures: at 20°C, its growth rate drops to 67% of its rate at 25°C, and it declines even more sharply at 15°C (7, 8). Lower temperature also inhibits the growth of this strain under photoautotrophic conditions; for example, the growth rate at 23°C is 69% that at 30°C, with biomass production following the same pattern (9). The narrow temperature range and specific seasonal conditions required for optimal growth and productivity of *E. gracilis* strain Z limit its geographic range. Developing cold-tolerant strains has emerged as an important strategy to overcome these limitations and unlock the full biomass potential of *E. gracilis*.

As part of the participatory science project “Everyone’s Euglena Project” (https://euglena-lab.com/report/minmide-pj-results-report/, accessed on 15 May 2025), we found 80 *E. gracilis* ecotypes in fresh water from all over Japan. After low temperature culture screening, we focused on *E. gracilis* ecotypes min34 and min41 with low-temperature resistance. These ecotypes, which were discovered in a cold region of northern Japan, are genetically distinct from previously identified strains based on the internal transcribed spacer 2 (ITS2) sequencing. Here, we demonstrated that strains min34 and min41 outperformed the industrial strain Z in terms of both growth rate and biomass productivity when cultured at 15°C, demonstrating an innate tolerance to low-temperature conditions. Additionally, we compared the paramylon, lipid, and carotenoid productivity of strains min41 and Z. Strain min41 showed superior productivity at low temperatures, paving the way for the expansion of *E. gracilis* cultivation to regions of lower temperature.

## RESULTS AND DISCUSSION

### Screening for low temperature-tolerant *Euglena*

We here selected 15°C as the low-temperature stress condition based on the finding that the growth of strain Z is significantly suppressed at this temperature (7). Although actual strain Z biomass production is carried out in the southernmost region of Japan (Okinawa) and Southeast Asia, where the culture temperature is maintained at 24–30°C, the pond water temperature in northern temperate regions generally drops to 15°C or less in winter.

To identify low-temperature-tolerant *Euglena*, we screened 80 strains obtained through the participatory science project. Each strain was autotrophically cultured in a 96-well plate at 15°C for 7 days. Twelve strains showed high OD_860_ values (Fig. S1) and were selected for further analysis. These 12 strains were then tested under mixotrophic culture conditions in flasks. Among them, two strains, min34 and min41, isolated from freshwater in northern Japan (Niigata and Iwate, respectively), exhibited particularly high cell density (Fig. S2), and were, therefore, selected as low-temperature-tolerant *Euglena*.

### Strains min34 and min41 are new isolates of *E. gracilis*

**Fig 1:**
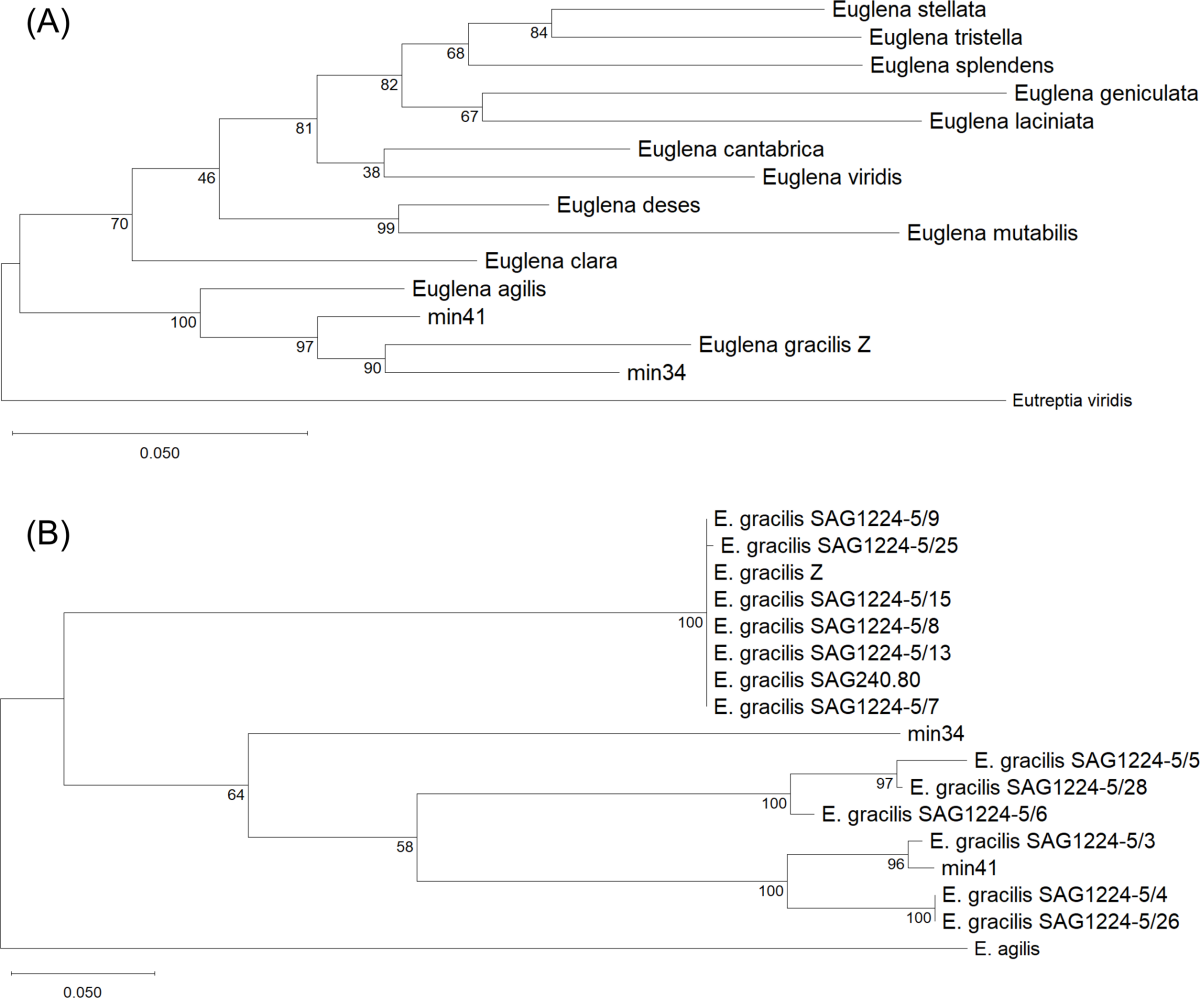
Phylogenetic trees of 16S rRNA (**A**) and ITS2 (**B**) sequences from strains min34, min41, and other *Euglena* species. *Eutreptia viridis* 16S rRNA and *Euglena agilis* ITS2 sequences were designated as the outgroups in the phylogenetic trees of 16S rRNA and ITS2, respectively. Numbers on the branches indicate bootstrap values. Scale bars represent the number of substitutions per site. The accession numbers of each sequence are listed in Table S3.

To examine the phylogenetic relationships of strains min34 and min41 with known euglenophytes, we analyzed their chloroplast 16S rRNA genes, a well-established genetic marker for species identification (10). As shown in Fig. 1A, strains min34 and min41 are phylogenetically closer to *E. gracilis* strain Z than to *E. agilis*, being located in the same clade as strain Z among the euglenophytes evaluated. ITS2 sequences are commonly used to assess the evolutionary relationships of *E. gracilis* strains (11). Therefore, we next examined the ITS2 sequences of min34, min41, and 14 other known *E. gracilis* strains. As shown in Fig. 1B, strain min34 formed an independent *E. gracilis* clade. Although min41 was most closely related to *E. gracilis* strain SAG1224-5/3 (Fig. 1B), their sequences were not identical (Fig. S1). These findings led us to categorize min34 and min41 as a newly isolated *E. gracilis* strains from the northern region of Japan.

### Strain min41 is more tolerant to low temperature

**Fig 2:**
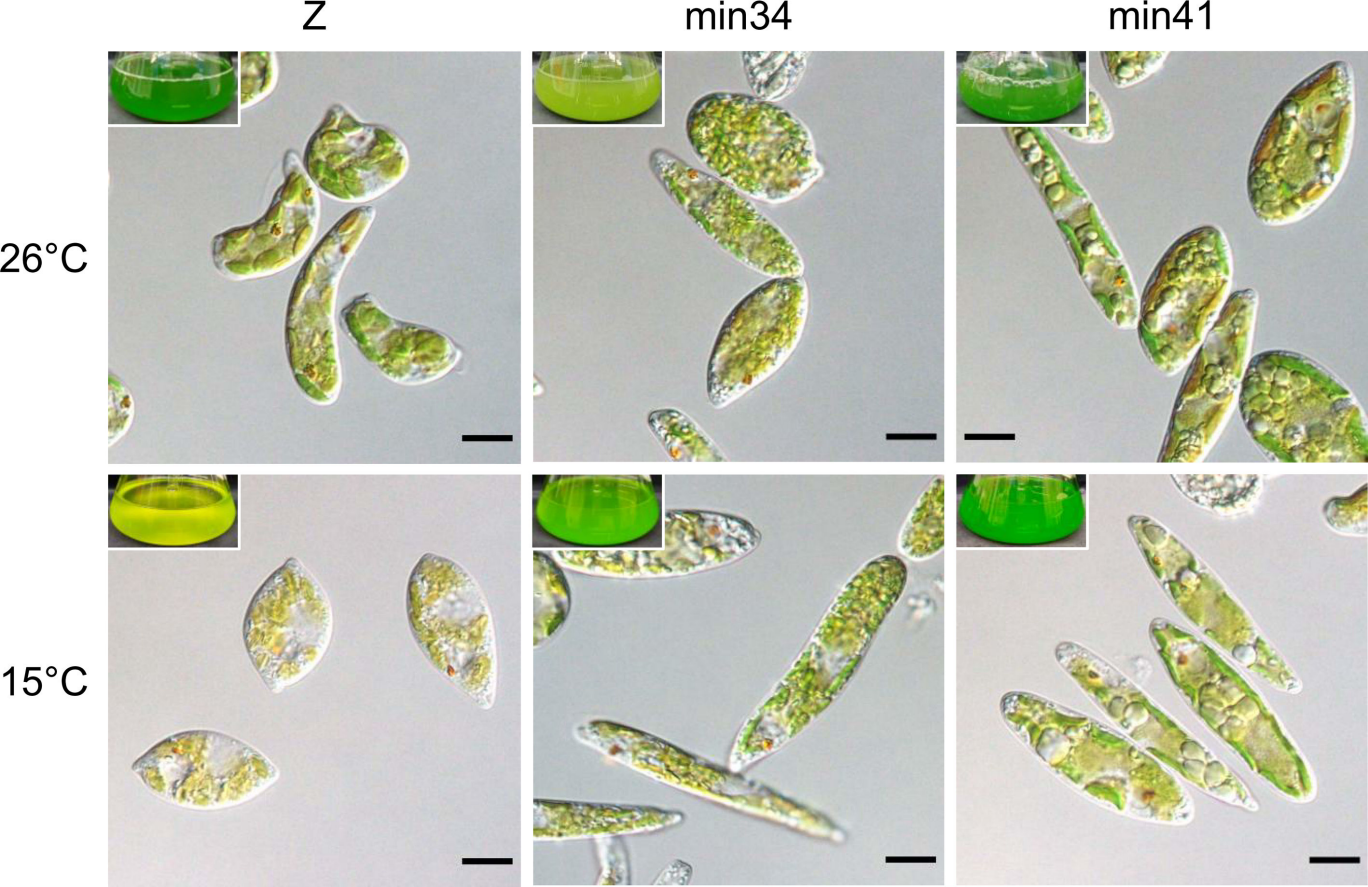
Microscopy and flask images of strains Z, min34, and min41 cultured at 26°C and 15°C. Scale bars are 10 µm.

To assess the sensitivity to low-temperature stress, we autotrophically cultured strains Z, min34, and min41 at 26°C and 15°C. This growth test differs from the secondary screening conditions, in which ethanol was supplemented as a carbon source. At 26°C, there was no apparent difference in cell appearance or culture color between strains Z and min41, whereas culture color greening of strain min41 was weakened. At 15°C, the cells and culture of strain Z appeared pale green, whereas no such change was observed in strains min34 and min41 (Fig. 2). The color change in strain Z at 15°C and strain at 26°C was confirmed by measuring chlorophyll content. At 26°C, strains Z and min41 exhibited similar chlorophyll contents on a per-cell basis, whereas the chlorophyll content in strain min34 was lower (Fig. S3). At 15°C, the chlorophyll content in strain Z decreased to 39% of the level at 26°C, whereas that of strain min41 remained stable. The chlorophyll content of strain min34 at 15°C was improved compared to 26°C, suggesting possible sensitivity of strain min34 at 26°C.

**Fig 3:**
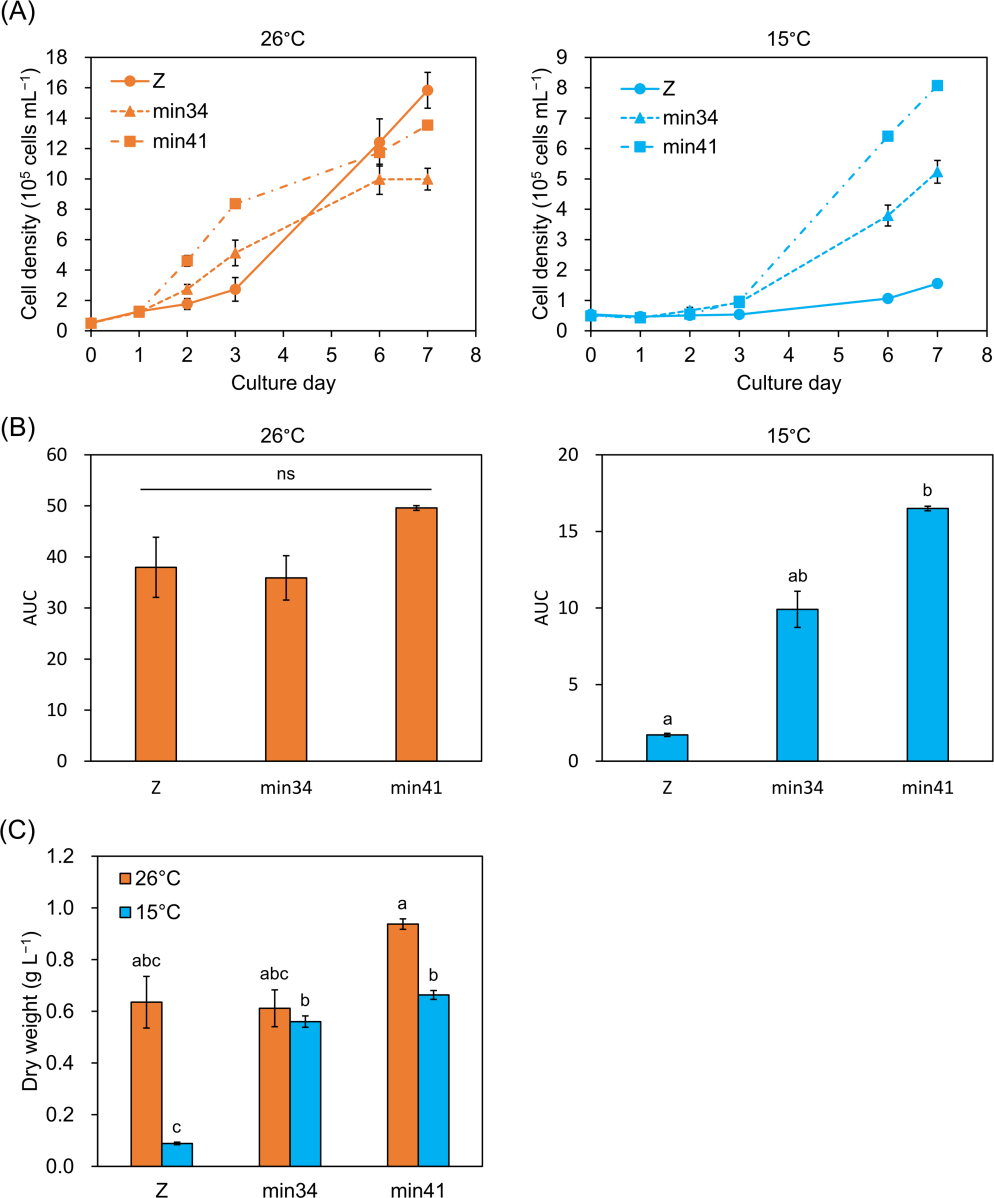
Growth properties of strains Z, min34, and min41 cultured at 26 and 15°C. (**A**) Growth curves at 26°C (left) and 15°C (right). (**B**) Area-under-the curve (AUC) analysis of growth curves 26°C (left) and 15°C (right). (**C**) Biomass productivity after 7 days of culture. Values are presented as the mean ± SE (*n* = 3). Values with different letters are significantly different according to the Welch’s one-way ANOVA followed by Welch’s *t*-test with Holm’s correction for multiple comparisons (*P* < 0.05). ns, not significant.

Next, we evaluated the growth kinetics of each strain at 26°C and 15°C (Fig. 3A). At 26°C, the cell densities of all strains initially increased 2.5-fold within the first day of culture. Subsequently, strain min41 exhibited the most rapid growth compared to other strains although strains Z and min41 ultimately reached comparable cell densities (1.4-1.6 × 10^6^ cells mL^−1^) after 7 days. The growth of strain min34 plateaued earlier than the other strains, eventually reaching 1.0 × 10^6^ cells mL^−1^. By contrast, at 15°C, strain Z exhibited severely inhibited growth for the first three days, followed by a slight increase. Strain min41 showed little increase in growth within the first day of culture but subsequently achieved a final cell density 5.2-times higher than that of strain Z. Strain min34 exhibited growth kinetics intermediate between strains Z and min41. To comprehensively evaluate cell growth over the entire culture period, we performed an area-under-the-curve (AUC) analysis. While AUC values at 26°C did not differ significantly among strains, the AUC at 15°C was 9.6-times higher in strain min41 than in strain Z, with strain min34 showing an intermediate value between strains min41 and Z (Fig. 3B).

These results suggest that min41 maintains robust growth even at 15°C, a temperature at which strain Z exhibits substantially impaired growth. During the seven-day cultivation period, strain min41 outperformed strain Z at both temperatures in terms of dry biomass. At 26°C, the biomass productivity of strain min41 was 1.5-fold greater than that of Z (Fig. 3C). This productivity gap widened significantly at 15°C, where min41 achieved a remarkable 7.5-fold higher dry weight compared to strain Z. The biomass productivity of strain min34 was also significantly higher than that of strain Z at 15°C, but slightly lower than that of strain min41. These results demonstrate that strain min41 produces markedly more biomass than strain Z, especially under low-temperature conditions. In addition, strain min41 consistently exhibited higher endpoint cell density, dry biomass, and chlorophyll content than strain min34 at both 26°C and 15°C. Based on these superior growth characteristics, we focused on the comparison of strain min41 with strain Z for further analyses.

Although strain min34 grew more slowly at 15°C than at 26°C (Fig. 3A), its total chlorophyll content at 15°C was 1.9-fold higher (Fig. S3). Because chlorophyll-mediated light harvesting is essential for photoautotrophic growth, this result may appear counterintuitive. However, an increase in chlorophyll levels at low temperature can be interpreted as a compensatory response to reduced activities of photosynthetic enzymes. Indeed, cold-adapted snow alga *Chlamydomonas nivalis* exhibits severely suppressed growth at 4°C, it nevertheless maintains high chlorophyll content and photosynthetic activity (12). The finding that different *E. gracilis* strains exhibit distinct low-temperature responses is, therefore, intriguing from the perspective of intraspecific diversity.

Our findings reveal that strain min41 is not only remarkably resilient to lower temperature conditions, but it also significantly outperforms strain Z in terms of biomass productivity under such conditions. This resilience is particularly noteworthy given that min41 was isolated from Iwate, a region in Japan that experiences low temperatures. The annual average temperature in Iwate is 12°C lower than in Okinawa (Japan Meteorological Agency, https://www.jma.go.jp/jma/indexe.html), a warmer region in Japan where strain Z is commercially produced for biomass. These findings suggest that strain min41 is naturally adapted to thrive in colder environments, thereby potentially expanding the scope for large-scale, outdoor cultivation of *E. gracilis* across diverse climatic zones. Such natural adaptability is reminiscent of other cold-resistant microalgal strains, such as *Chlorella vulgaris* 13-1, which was isolated from the polar regions of northern Sweden and has comparable biomass productivity at 5°C and 25°C (13, 14). Collectively, our findings highlight the importance of targeted isolation of microalgae ecotypes from various climatic regions to optimize biomass production across a broader temperature range.

### Strain min41 exhibits excellent bioproduction under low-temperature conditions

**Fig 4:**
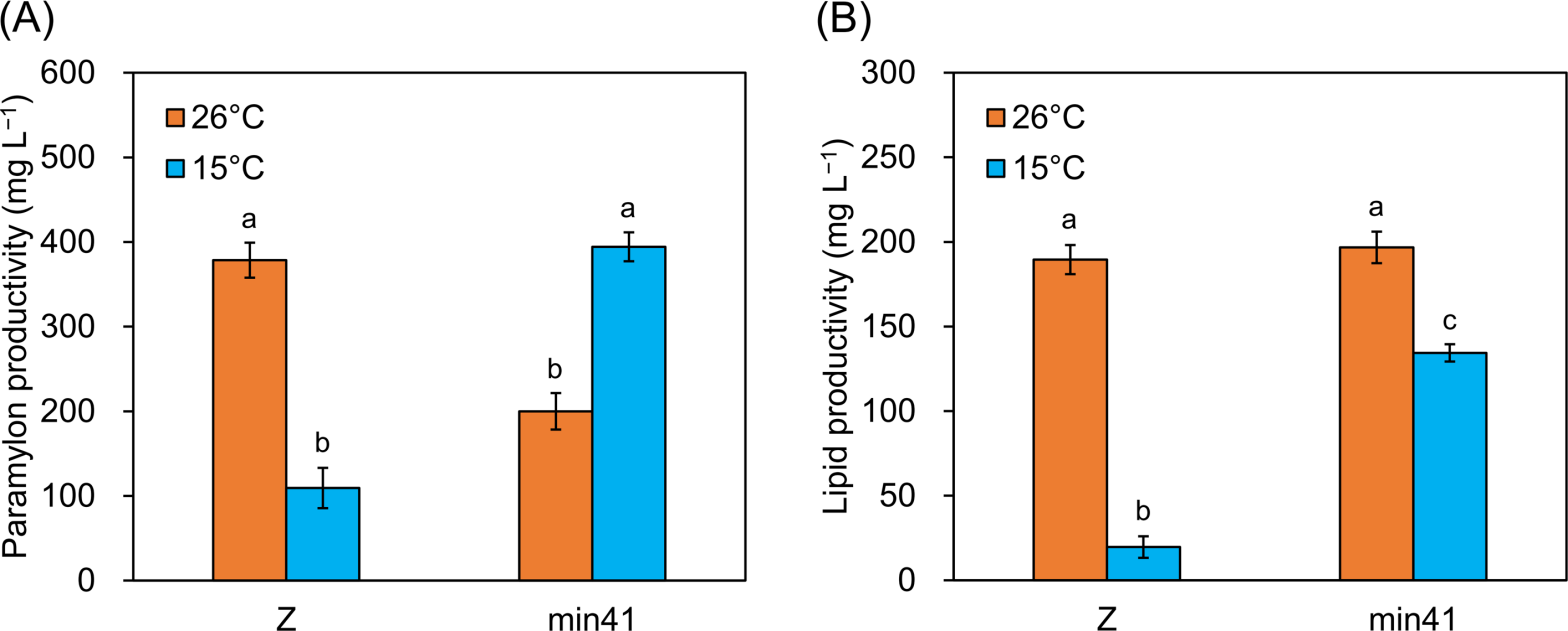
Paramylon and lipid productivity of strains Z and min41 cultured at 26°C and 15°C. (**A**) Paramylon productivity. Cells were autotrophically cultured at each temperature for 7 days and incubated with shaking in nitrogen-depleted medium for 3 days (aerobic). (**B**) Lipid productivity. After nitrogen depletion treatment, cells were incubated without shaking for 2 days in the dark (hypoxic). Values are presented as the mean ± SE (*n* = 3). Values with different letters are significantly different according to the Welch’s one-way ANOVA followed by Welch’s *t*-test with Holm’s correction for multiple comparisons (*P* < 0.05). All data for paramylon productivity after hypoxic treatment and lipid productivity after aerobic treatment are shown in Table S1 and S2.

To further evaluate the potential of strain min41 as a biomass resource, we examined the productivity of representative high-value compounds produced by *E. gracilis*: paramylon, lipids, and carotenoids. At 26°C, the paramylon productivity of strain min41 was 53% that of strain Z, and its lipid productivity was comparable to that of strain Z (Fig. 4; Table S1 and S2). However, at 15°C, strain min41 exhibited 3.6- to 6.8-fold higher productivity of paramylon and lipids compared to strain Z. When comparing the effects of temperature on productivity, strain min41 showed substantially higher paramylon productivity at 15°C than at 26°C; however, the subsequent hypoxic conversion of paramylon into lipids did not increase proportionally. This discrepancy may reflect a temperature sensitivity in the enzymatic steps involved in paramylon degradation and wax ester synthesis, resulting in reduced efficiency of lipid accumulation at low temperature. Consistent with this idea, the cellular lipid content of strain Z cultured at 15°C remained almost unchanged between aerobic and hypoxic conditions, further supporting the notion that wax ester synthesis is strongly constrained at low temperature.

**Fig 5:**
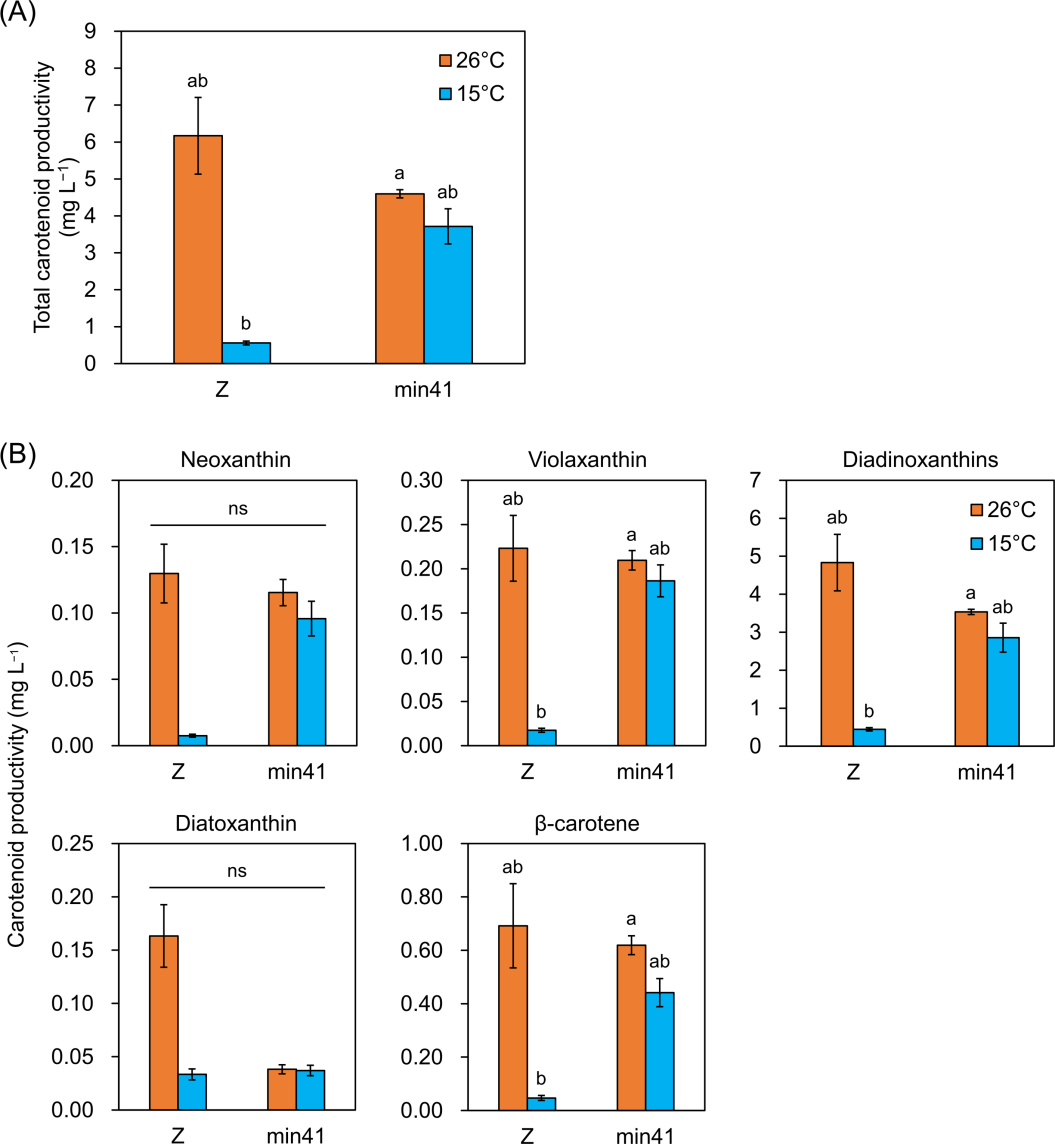
Carotenoid productivity of strains Z and min41 cultured at 26°C and 15°C. Cells were autotrophically cultured at each temperature for 7 days. (**A**) Total carotenoid productivity. (**B**) Productivity of each carotenoid. Values are presented as the mean ± SE (*n* = 3). Values with different letters are significantly different according to the Welch’s one-way ANOVA followed by Welch’s *t*-test with Holm’s correction for multiple comparisons (*P* < 0.05). ns, not significant.

Similar to paramylon and lipid productivity, the total carotenoid productivity of strain min41 was 75% that of strain Z at 26°C, whereas at 15°C, it was 6.7-times higher than that of strain Z (Fig. 5A). The productivity of the carotenoids neoxanthin, violaxanthin, diadinoxanthin, and β-carotene was also enhanced in strain min41, ranging from 6.4- to 12.7-fold greater than that of strain Z at 15°C (Fig. 5B). These results demonstrate that strain min41 exhibits excellent bioproduction under low-temperature conditions.

Consistent with these findings, Yuan et al. (15) reported that low-temperature treatment (16°C and 4°C) of *E. gracilis* strain Z inhibited growth and reduced the levels of various metabolites, including chlorophyll a, carotenoids, and lipids (15). Although the authors observed an increase in paramylon content under low-temperature conditions, we observed a decrease in the current study. However, our experimental setup involved nitrogen-depleted medium, which is different from their experimental conditions. The authors also showed that low temperature pretreatment helped alleviate subsequent low-temperature stress. Although our study did not include a low temperature pretreatment, it may be worthwhile to investigate the effects of such a treatment on various *E. gracilis* ecotypes, including min41. Pretreatment of min41 might further enhance its biomass productivity at 15°C. Such analysis might also help determine whether the low temperature tolerance of this strain is constitutive or environmentally inducible.

In the current study, no significant differences in the levels of the carotenoid diatoxanthin were observed between strains Z and min41 at 15°C (Fig. 5B). In detail, the relative diatoxanthin content per cell in strain Z increased from 2.7% at 26°C to 6.1% at 15°C, whereas that of strain min41 remained nearly constant (0.8%–1.0%) across both temperatures. Thus, strain Z responded to low temperature by increasing its diatoxanthin content, while min41 showed no such temperature-dependent change. This difference accounts for the comparable diatoxanthin productivity observed between the two strains (Fig. 5B). The increased diatoxanthin accumulation in strain Z may contribute to protection against prolonged low-temperature stress. This idea is supported by the finding that diatoxanthin treatment alleviates UV-induced oxidative damage more effectively than other carotenoids in mammalian cells (16). In line with this, a previous study reporting diatoxanthin accumulation in strain Z under low-temperature and high-light conditions further supports this idea (7). Furthermore, the paramylon dynamics of strain min41 were markedly different from those of strain Z at both temperatures, with paramylon accumulation being more strongly induced at 15°C (Fig. 4A). As reported in *Chlamydomonas reinhardtii*, where starch accumulation was induced by treatment at 5°C (17), paramylon accumulation in strain min41 may represent one of its low temperature adaptation strategies. Although it remains unclear whether paramylon itself directly contributes to low-temperature stress protection, various carbohydrates, including trehalose, are known to mitigate cold stress. Trehalose, which is synthesized in *E. gracilis* (18), functions as a chemical chaperone that stabilizes proteins and lipid bilayers (19, 20). Low-temperature stress can also trigger osmotic stress, and *E. gracilis* responds to such conditions by degrading paramylon and markedly accumulating trehalose (18). Thus, dynamic remodeling of carbon flux from accumulated paramylon toward protective sugars may contribute to the low temperature adaptation of strain min41.

In conclusion, through our participatory science project, we successfully isolated new strains of *E. gracilis* named min34 and min41. Especially, strain min41 is most adapted to low-temperature conditions (15°C), thereby distinguishing it from the commonly used strain Z. This makes min41 a promising candidate for outdoor biomass production in colder climates. Notably, min41 exhibited higher productivity of paramylon, lipids, and carotenoids under low-temperature conditions. These findings point to the genetic diversity and environmental adaptability among *E. gracilis* ecotypes and highlight the potential value of strain min41 as a genetic resource for expanding the applicability of outdoor mass cultivation for biomass production.

## MATERIALS AND METHODS

### Strains and culture conditions

*E. gracilis* strain Z (NIES-48) was originally provided by the Institute of Applied Microbiology collection, the University of Tokyo (Tokyo, Japan), and *E. agilis* (NIES-3945) is available from the National Institute for Environmental Studies (Tsukuba, Japan). *E. gracilis* SAG strains (strain numbers: 1224-5/3, 1224-5/4, 1224-5/5, 1224-5/6, 1224-5/7, 1224-5/8, 1224-5/9, 1224-5/13, 1224-5/15, 1224-5/25, 1224-5/26, 1224-5/28, and 240.80) were provided by the Culture Collection of Algae at Göttingen University (Göttingen, Germany). Strains were maintained in citrate-free CM (modified CM) (21) medium (pH 3.5) containing 0.1% (vol/vol) ethanol under 50 μmol photons m^−2^ s^−1^ light (12-h:12-h light-dark cycle) at 29°C on a rotary shaker (120 rpm). The light intensity was measured using a HD2302.01 portable luxmeter and an LP471PAR probe (400–700 nm; Delta Ohm, Veneto, Italy).

### Screening for low temperature-tolerant *Euglena* strains

The 80 *Euglena* strains were obtained through the participatory science project, which involved collecting freshwater samples across Japan. The freshwater sample was cultured in modified CM medium (pH 3.5) for 2–3 weeks. Cells were sorted into a 96-well plate, with each well containing 200 μL of modified CM:KH (4:1, vol/vol) (22) medium (pH 3.5), using a MoFlo XDP fluorescence-activated cell sorter (Beckman Coulter, CA, United States) as described previously (23). An initial screening was conducted in 96-well plate. Each well contained 200 μL of modified CM medium (pH 3.5), and cultures were incubated at 15°C under continuous illumination at 50 μmol photons m^−2^ s^−1^ for 7 days without shaking. Growth was monitored by measuring optical density at 860 nm (OD_860_) on days 0 and 7. Based on the results of this preliminary screening, 12 strains exhibiting higher OD_860_ values were subjected to a secondary screening. In this step, each selected strain was cultured in 100-mL Erlenmeyer flasks containing 50 mL of modified CM medium (pH 3.5), supplemented with 0.1% (vol/vol) ethanol as carbon source. Cultures were incubated under the same temperature and light conditions (15°C, 50 μmol photons m^−2^ s^−1^, continuous light) and shaken at 120 rpm. Cell density was measured with a particle analyzer (CDA-1000, Sysmex, Kobe, Japan).

### Phylogenetic analysis

Genomic DNA was extracted from each *Euglena* strain cultured in modified CM medium (pH 3.5) using a Kaneka Easy DNA Extraction Kit v.2 (Kaneka, Osaka, Japan). DNA fragments were amplified with GoTaq DNA Polymerase (Promega, WI, United States) using a specific primer set for 16S rRNA (forward, 5′-TTGATCCTGGCTCAGGATGAACGCT-3′, position 15-39; reverse, 5′-CAAGGAGGTGATCCAGCCGCACCTT-3′, position 1446-1470) and ITS2 (forward, 5′-TTCTGAGGAAGGACACAGCAGC-3′; reverse, 5′-TTCCTCCACTGAGTGATATGC-3′). The ITS2 forward and reverse primers anneal within LSU rRNA species 1 and species 2, respectively, based on the *E. gracilis* rRNA gene (accession no. X53361). The PCR products were cleaned using ExoSAP-IT Express PCR Product Cleanup Reagent (Thermo Fisher Scientific, MA, United States) and subjected to Sanger sequencing. The ITS2 region was sequenced using the same primer pair as in PCR. Because the chloroplast 16S rRNA region exceeded the read length of Sanger sequencing, two additional internal primers, 647-F (5′-ATTTCCAGTGTAGCGGTG-3′) and 718-R (5′-ACTTAGTATCCATAGTTTACG-3′), were designed for sequencing. The 5′ portion of the 16S rRNA fragment was sequenced using 16S-F together with 647-F, and the 3′ portion was sequenced using 718-R together with 16S-R. The internal primers were designed to generate overlapping regions between reads. All bidirectional reads were inspected, trimmed, and assembled into a single consensus 16S rRNA sequence for each strain based on the overlapping regions. The 16S rRNA sequence of strain min41 and the ITS2 sequences of strains Z, min41, SAG series, and *E. agilis* were newly sequenced in this study. Multiple sequence alignment was performed using MUSCLE implemented in MEGA 12 with default parameters. Phylogenetic analysis was conducted using the Maximum Likelihood method based on the Tamura-Nei model, and node support was assessed with 1,000 bootstrap iterations.

### Growth test

The *E. gracilis* cells were inoculated at an initial cell density of 5 × 10^4^ cells mL^−1^ in modified CM medium (pH 3.5) and cultured for 7 days on a rotary shaker (120 rpm) at 15°C and 26°C under continuous illumination (50 μmol photons m^−2^ s^−1^). For CO_2_ supplementation, a 5% CO_2_–air mixture was delivered by direct bubbling into the culture medium at a flow rate of 120 mL min^−1^. Cell density was measured as described above. AUC was obtained by subtracting a fixed baseline value of 0.5, corresponding to the initial cell density of 5 × 10^4^ cells mL^−1^, from each measurement and integrating the resulting values over time using the trapezoidal rule. After 7 days of culture, the cells were harvested by centrifugation using a swing rotor at 2,800 × *g* for 5 min. After centrifugation, the supernatant was completely removed, and the cell pellets were immediately frozen at −80°C. For pigment quantification, the frozen pellets were used as described below. To measure biomass productivity, the harvested cells were subsequently dried in a freeze dryer (FDV-1200, EYELA, Tokyo, Japan).

### Microscopy

Microscopy images were obtained using an Olympus BX51 upright microscope with a 100× oil-immersion objective lens (Olympus, Tokyo, Japan). Differential interference contrast (DIC) imaging was performed under epi-illumination using a halogen lamp as the light source.

### Pigment quantification

Chlorophyll extraction and measurement were performed as described previously (24). The absorbances were measured using a UV-1900i spectrophotometer (Shimadzu, Kyoto, Japan). Carotenoid extraction and LC/MS analysis were performed as previously described (25).

### Paramylon and lipid quantification

One-week-old *E. gracilis* cultures grown as described above were harvested by centrifugation. The medium was replaced with nitrogen-free modified CM medium (pH 3.5), and the cells were cultured under the same conditions as above for 3 days (aerobic samples). This treatment promotes paramylon accumulation without a carbon source. The remaining culture was transferred to a 50 mL tube, sealed, and incubated in the dark without shaking for 2 days (hypoxic sample). Cells subjected to aerobic and hypoxic treatments were harvested by centrifugation and dried using a freeze dryer. Paramylon and lipid contents were analyzed as described previously (26).

### Statistical analysis

All data are presented as mean ± SE (*n* = 3). Statistical analyses were performed using Welch’s one-way analysis of variance (ANOVA). When significant differences were detected, post hoc pairwise comparisons were conducted using Welch’s *t*-test with Holm’s correction for multiple comparisons. Differences were considered statistically significant at *P* < 0.05.

## Data Availability

The accession numbers of the 16S rDNA and ITS2 sequences used for phylogenetic analysis are listed in Table S3. The data underlying this article are available in the article and in its Supplemental material.

## SUPPLEMENTAL MATERIAL

Supplemental material aem.00195-26-s0001.pdf Fig. S1 to S3; Tables S1 to S3.

ASM does not own the copyrights to Supplemental Material that may be linked to, or accessed through, an article. The authors have granted ASM a non-exclusive, world-wide license to publish the Supplemental Material files. Please contact the corresponding author directly for reuse.
