# Supplementary material for: A low temperature-adapted Euglena gracilis ecotype for outdoor biomass production in colder climates
Source: Appl Environ Microbiol. 2026 May 4;92(5):e00195-26. doi: 10.1128/aem.00195-26 (PMC13188879; doi:10.1128/aem.00195-26)
Supplement: Supplemental material — Fig. S1 to S3; Tables S1 to S3. [file aem.00195-26-s0001.pdf]

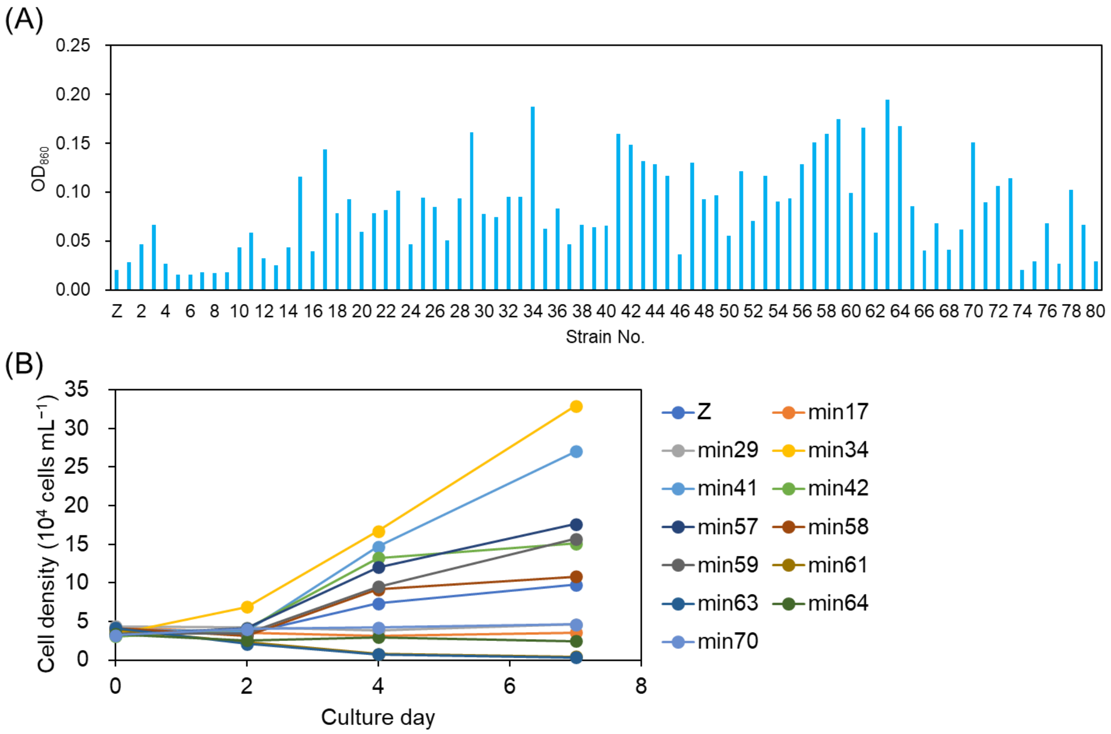

Supplementary Figure S1. Screening for low temperature-tolerant *Euglena* strains. (A) OD_{860} measurements on day 7 of preliminary screening conducted in 96-well plates. A total of 80 *Euglena* strains, including the standard strain Z, were cultured at 15°C under continuous light in modified CM medium (pH 3.5) without shaking. Each bar represents the OD860 value of an individual strain (*n*=1). (B) Growth curves of selected 12 *Euglena* strains in flask cultures. Each strain was cultured in 50 mL of modified CM medium (pH 3.5) supplemented with 0.1% (v/v) ethanol in 100-mL Erlenmeyer flasks at 15°C under continuous light. Cultures were shaken at 120 rpm but without aeration. Cell density was monitored over a 7-day period (*n*=1).

```
SAG1224-5/3 CTGGACAAAGGAACAGCTCTGAACGCAACTGGCCAGCAAGGGGTACCACCCCAAGCTGCC   60
min41       CTGGACAAAGGAACAGCTCTGAACGCAACTGGCCAGCAAGGGGTACCACCCCAAGCTGCC   60

SAG1224-5/3 TGTGCGCCAGTGTTGGCTTGATGGGAGCAGGTCGCAGCAGCAGCCCTCCCCAGTCTCTGG  120
min41       TGTGCGCCAGTGTTGGCTTGATGGGAGCAGGTCGCAGCAGCAGCCCTCCCCAGTCTCTGG  120

SAG1224-5/3 TGTGTGGTGACTCTCTCGGCCTCTCCCAGTGCGATCGATGACCGGCGGTGGTATGATGCT  180
min41       TGTGTGGTGACTCTCTCGGCCTCTCCCAGTGCGATCGATGACCAGCGGTGGTATGATGCT  180

SAG1224-5/3 TGT--CTCCCACGGCGAGTCCGTGCCATTACTGGTTCCTTGTTGTTGTGGCTAATGTCGG  238
min41       TGTCACTCCCACGGCGAGTCCGTGCCATTGCTGGTTCCTTGTTGTTGTGGCTAATGTCGG  240

SAG1224-5/3 CTGGAATGTCTGTCCGAGCGTGGGTCCCAGGGCTCGCGCTCCCGTGCTGATGGCTGAAAC  298
min41       CTGGAATGTCTGTCCGAGCGTGGGTCCCAGGGCTCACGCTCCCGTGCTGATGGCTGAAAC  300

SAG1224-5/3 TTGTGGTCAACGTCCTGGAGGGAGCAGGCACCAGGAACCCAACAACGCATCTGCCTCACA  358
min41       TTGTGGTCAACGTCCTGGAGGGAGCAGGCACCAGGAACCCAACAACGCATCTGCCTCACA  360

SAG1224-5/3 CCGAGCGGCTTCTCCAGTGGCATTTCGGCCCGAATCGGGC---AACGTTGCCCATTGGCT  415
min41       CCGAGTGGCTTCTCCAGTGGCATTTCGGCCCGAATCGGGCAACAACGTTGCCCATTGGCT  420

SAG1224-5/3 CTTGTGACCTGGCGC                                               430
min41       CTTGTGACCTGGCGC                                               435
```

Supplementary Figure S2. Alignment of ITS2 sequences from *Euglena gracilis* strains SAG1224-5/3 and min41. White letters on a black background indicate different bases.

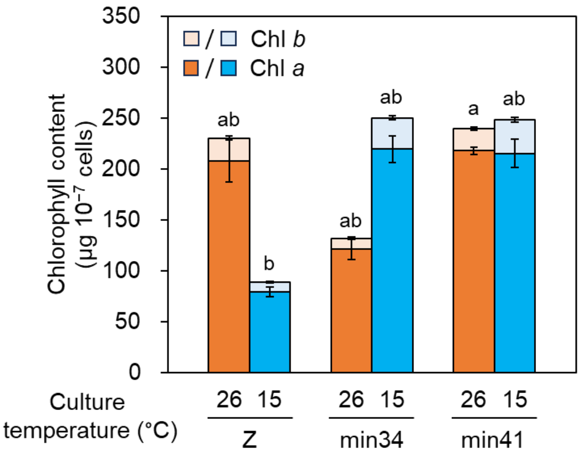

Supplementary Figure S3. Total chlorophyll content and composition after 7 days of culture. Values are presented as the mean ± SE (*n*=3). Values with different letters are significantly different according to the Welch's one-way ANOVA followed by Welch's *t*-test with Holm's correction for multiple comparisons ($p < 0.05$).

Supplementary Table S1. Paramylon productivity of strains Z and min41 cultured at 26°C and 15°C. Cells were autotrophically cultured at each temperature for 7 days. Aerobic samples were collected after 3 days of incubation with shaking in nitrogen-depleted medium. Hypoxic samples were collected after an additional 2 days of incubation without shaking in the dark. All treatments were maintained at each temperature. Values are presented as the mean ± SE (n=3). Statistical analyses were conducted separately under aerobic and hypoxic conditions using Welch's one-way ANOVA followed by Welch's t-test with Holm correction. Different letters indicate statistically significant differences (p < 0.05). ns, not significant.

Temperature	26°C		15°C	
Strain	Z	min41	Z	min41
	(mg L^{-1})			
Aerobic	378.5±20.7^{a}	199.9±21.6^{b}	109.3±23.8^{b}	394.3±17.1^{a}
Hypoxic	30.8±3.9^{ns}	8.9±1.8^{ns}	62.1±9.2^{ns}	198.0±19.6^{ns}

Supplementary Table S2. Lipid contents of strains Z and min41 cultured at 26°C and 15°C. Cells were autotrophically cultured at each temperature for 7 days. Aerobic samples were collected after 3 days of incubation with shaking in nitrogen-depleted medium. Hypoxic samples were collected after an additional 2 days of incubation without shaking in the dark. All treatments were maintained at each temperature. Values are presented as the mean ± SE (n=3). Statistical analyses were conducted separately under aerobic and hypoxic conditions using Welch's one-way ANOVA followed by Welch's *t*-test with Holm correction. Different letters indicate statistically significant differences ($p < 0.05$).

Temperature	26°C		15°C	
Strain	Z	min41	Z	min41
		(mg L^{-1})		
Aerobic	70.4±3.8^{a}	105.8±2.5^{b}	17.5±3.6^{c}	72.0±9.8^{abc}
Hypoxic	189.5±8.6^{a}	196.7±9.3^{a}	19.6±6.4^{b}	134.4±5.1^{c}

Supplementary Table S3. List of accession numbers of the sequences used for phylogenetic tree construction.

Sequence	Species and strain	Accession number
16S rRNA	*Euglena gracilis* Z	V00159
	Euglena gracilis min34	LC891937
	Euglena gracilis min41	LC875782
	Euglena agilis	FJ719652
	Euglena cantabrica	AY626047
	Euglena clara	FJ719654
	Euglena deses	AY626043
	Euglena geniculata	AY070252
	Euglena laciniata	EU221487
	Euglena mutabilis	AY626044
	Euglena splendens	FJ719662
	Euglena stellata	EU221493
	Euglena tristella	FJ719664
	Euglena viridis	FJ719665
	Eutreptia viridis	FJ719670
ITS2	*Euglena gracilis* Z	LC877795
	Euglena gracilis min34	LC891938
	Euglena gracilis min41	LC877796
	Euglena gracilis SAG1224-5/3	LC877797
	Euglena gracilis SAG1224-5/4	LC877798
	Euglena gracilis SAG1224-5/5	LC877799
	Euglena gracilis SAG1224-5/6	LC877800
	Euglena gracilis SAG1224-5/7	LC877801
	Euglena gracilis SAG1224-5/8	LC877802
	Euglena gracilis SAG1224-5/9	LC877803
	Euglena gracilis SAG1224-5/13	LC877804
	Euglena gracilis SAG1224-5/15	LC877805
	Euglena gracilis SAG1224-5/25	LC877806
	Euglena gracilis SAG1224-5/26	LC877807
	Euglena gracilis SAG1224-5/28	LC877808

	Euglena gracilis SAG240.80	LC877809
	Euglena agilis	LC877810
